# IgE-Selective Immunoadsorption for Severe Atopic Dermatitis

**DOI:** 10.3389/fmed.2018.00027

**Published:** 2018-02-12

**Authors:** Michael Kasperkiewicz, Sophie-Charlotte Mook, Diana Knuth-Rehr, Artem Vorobyev, Ralf J. Ludwig, Detlef Zillikens, Philip Muck, Enno Schmidt

**Affiliations:** ^1^Department of Dermatology, University of Lübeck, Lübeck, Germany; ^2^Lübeck Institute of Experimental Dermatology, University of Lübeck, Lübeck, Germany; ^3^Department of Internal Medicine, University of Lübeck, Lübeck, Germany

**Keywords:** atopic dermatitis, IgE, immunoadsorption, immunoglobulin, SCORAD

## Abstract

**Introduction:**

Recent reports proposed the application of immunoadsorption (IA) for patients with recalcitrant atopic dermatitis (AD) and high-serum IgE levels. However, experience with this novel treatment approach, especially with the newly available IgE-specific adsorber, is limited and recommendation for its use in clinical practice awaits evidence from more studies.

**Materials and methods:**

Patients with severe AD (SCORAD ≥ 60) and total serum IgE levels ≥750 kU/L were included in this study. The treatment protocol consisted of two cycles of five consecutive treatments with IgE-selective IA 3 weeks apart.

**Results:**

Ten patients were enrolled and four patients completed the study. The mean SCORAD was significantly improved by up to 43% within a few weeks and until the end of a 6-month follow-up period, with 50% of patients achieving an at least 50% individual reduction of the baseline SCORAD. Each IA cycle induced a temporal average decrement of total serum levels of IgE, IgM, IgA, and IgG by 92, 43, 38, and 35%, respectively. Except for one case of *Staphylococcus aureus* septicemia, no major adverse events occurred.

**Conclusion:**

Although limited by a considerable withdrawal rate, our observations strengthen our and other recent results further suggesting that IgE-selective IA is an effective treatment option for patients severely affected by AD with highly elevated IgE levels.

## Introduction

Atopic dermatitis (AD) is characterized by pruritic, eczematous skin lesions, and is commonly associated with elevated serum IgE levels. AD can considerably impact the patient’s quality of life and is one of the most frequent chronic inflammatory cutaneous disorders. For patients who are severely affected by the disease, conventional immunosuppressive treatments may not show uniform efficacy and can be limited by severe side effects (1).

Although the role of IgE in the pathophysiology of AD is not fully understood, sequestering free IgE by the anti-IgE monoclonal antibody omalizumab showed some clinical benefit in AD patients with poor response to traditional therapy (2). However, studies of this treatment have yielded controversial results. A recent systematic review and meta-analysis of the efficacy of omalizumab in 103 AD patients from 13 studies revealed that serum IgE concentrations of >700 kU/L were associated with less-favorable clinical responses compared with lower levels (2). The limited effect of omalizumab in AD patients with high total IgE serum levels may be related to insufficient IgE neutralization by omalizumab. The recommended dosing table for omalizumab is limited to 150–1,200 mg/month, according to total IgE concentrations within a range between 30 and 1,500 kU/L (3).

While adjuvant immunoadsorption (IA) has been introduced in dermatology by its successful application in patients with severe and/or refractory pemphigus (4), its use in AD is still limited to individual centers. We and others, however, have recently demonstrated that IA may represent an alternative IgE depletion method in patients with very high IgE concentrations leading to normalization of cutaneous inflammation parameters and clinical improvement of AD (5–9). While panimmunoglobulin IA was initially employed, an IgE-specific adsorber column has more recently become available (6, 9). Since experience with this novel treatment approach in AD is limited and recommendation for its use in clinical practice will require more clinical data, we employed IgE-selective IA in a further series of patients with severe AD and considerably elevated serum IgE levels. For this group of patients, there still remains a large unmet medical need for powerful new treatment options.

## Materials and Methods

### Patients

In this study, 10 patients (seven males and three females, mean age 40.3 ± 18.7, range 18–70 years) with severe AD [mean SCORAD (SCORing AD index) 67.5 ± 5.4, range 61.5–81] and greatly elevated serum IgE levels (mean 5,377.7 ± 6,775.3 kU/L, range 931–21,510 kU/L) were enrolled at the Department of Dermatology of the University of Lübeck. The inclusion criteria were as follows: (1) severe AD, i.e., SCORAD ≥ 60, requiring systemic immunosuppressive treatment, (2) total serum IgE level ≥750 kU/L, and (3) ≥18 years of age. The exclusion criteria were as follows: (1) known hypersensitivity or allergy to materials used in the adsorber columns, (2) no possibility of an adequate anticoagulation (e.g., multiple allergies to various anticoagulants), (3) bleeding disorders including hypo- and hypercoagulabilities, (4) severe cardiovascular disease (cardiac failure, NYHA III and IV), (5) severe systemic infections extending beyond skin, (6) serum IgG level <250 mg/dL, (7) severe immunodeficiency (e.g., AIDS), (8) treatment with an angiotensin-converting enzyme inhibitor that could not be omitted 72 h before IA, and (9) pregnancy. The majority of patients had previously not adequately responded to or had side effects from treatment with systemic corticosteroids and/or cyclosporine A. Written informed consent was obtained from all patients before participation in this study, which was approved by the ethics committee of the University of Lübeck and followed the Declaration of Helsinki.

### Treatment

Two cycles of five IA sessions on days 1 to 5 (week 1) and days 29 to 33 (week 5) were performed as described previously (5, 6), except for one patient in whom the second cycle was split into two parts (2 × IA and 3 × IA 2 weeks apart) because of an adverse event. In each IA, plasma was separated using a blood cell separation technique (Life-18 Apheresis Unit; Miltenyi Biotec, Bergisch Glattbach, Germany) followed by alternate application of two patient plasma volumes to two adsorption columns (30–35 cycles of approximately 8,000 mL separated plasma) containing monoclonal mouse anti-human IgE (TheraSorb^®^-IgE; Miltenyi Biotec).

In half of the patients, a peripheral venous catheter was used, and in the other half, central venous access was required due to difficult peripheral veins. Patients receiving a central venous catheter were instructed to prophylactically apply topical fusidic acid to the neck area for 3 days before each IA cycle. Previous topical [corticosteroids (*n* = 9 patients in total; class I: *n* = 1, class II: *n* = 1, class III: *n* = 1, class IV: *n* = 3, class V: *n* = 2, class VII: *n* = 3), calcineurin inhibitors (*n* = 1; tacrolimus)] and/or systemic treatments [antihistamines (*n* = 5 patients in total; cetirizine 10–20 mg/day: *n* = 3, hydroxyzine 25 mg/day: *n* = 3, desloratadine 20 mg/day: *n* = 1, loratadine 20 mg/day: *n* = 1), corticosteroids (*n* = 1; prednisolone 10 mg/day), cyclosporine A (*n* = 0)] were initially continued without change of frequency and/or dosage and were subsequently allowed to be modified as needed.

### Clinical and Laboratory Examinations

Patients were prospectively assessed by dermatologists both as inpatients (on the days when IA was performed) and outpatients at the Department of Dermatology of the University of Lübeck. During a follow-up period of up to 6 months following IA (weeks 1, 3, 5, 9, 13, 17, and 25), the SCORAD index was applied to evaluate the clinical course of the patients. Additionally, the use of concomitant therapy for AD and adverse events were documented. Total levels of serum IgE, IgG, IgM, and IgA were measured before and after IA using the UniCAP system (Phadia; IgE) and the BN Prospec Nephelometer system (Siemens; IgG, IgM, and IgA) (5).

### Statistical Analyses

Data are presented as means ± SD (SCORAD) and box-and-whisker plots (serum immunoglobulins). Repeated measurements of SCORAD were compared with baseline values using one-way ANOVA, with *P* < 0.05 considered statistically significant.

## Results

### Clinical Course

Of the 10 patients enrolled, two patients withdrew from the study due to an adverse event (central venous catheter-associated *Staphylococcus aureus* septicemia and pain related to intravenous catheter insertion, respectively) during the first IA cycle. Two other patients dropped out because of a coping/compliance problem and absence from follow-up examinations in week 5, but were included in the analysis. Other adverse events included fatigue in one patient necessitating to split the second IA cycle into two parts and edema formation of the hands and feet in another patient. Otherwise, IA was relatively well tolerated.

**Figure 1:**
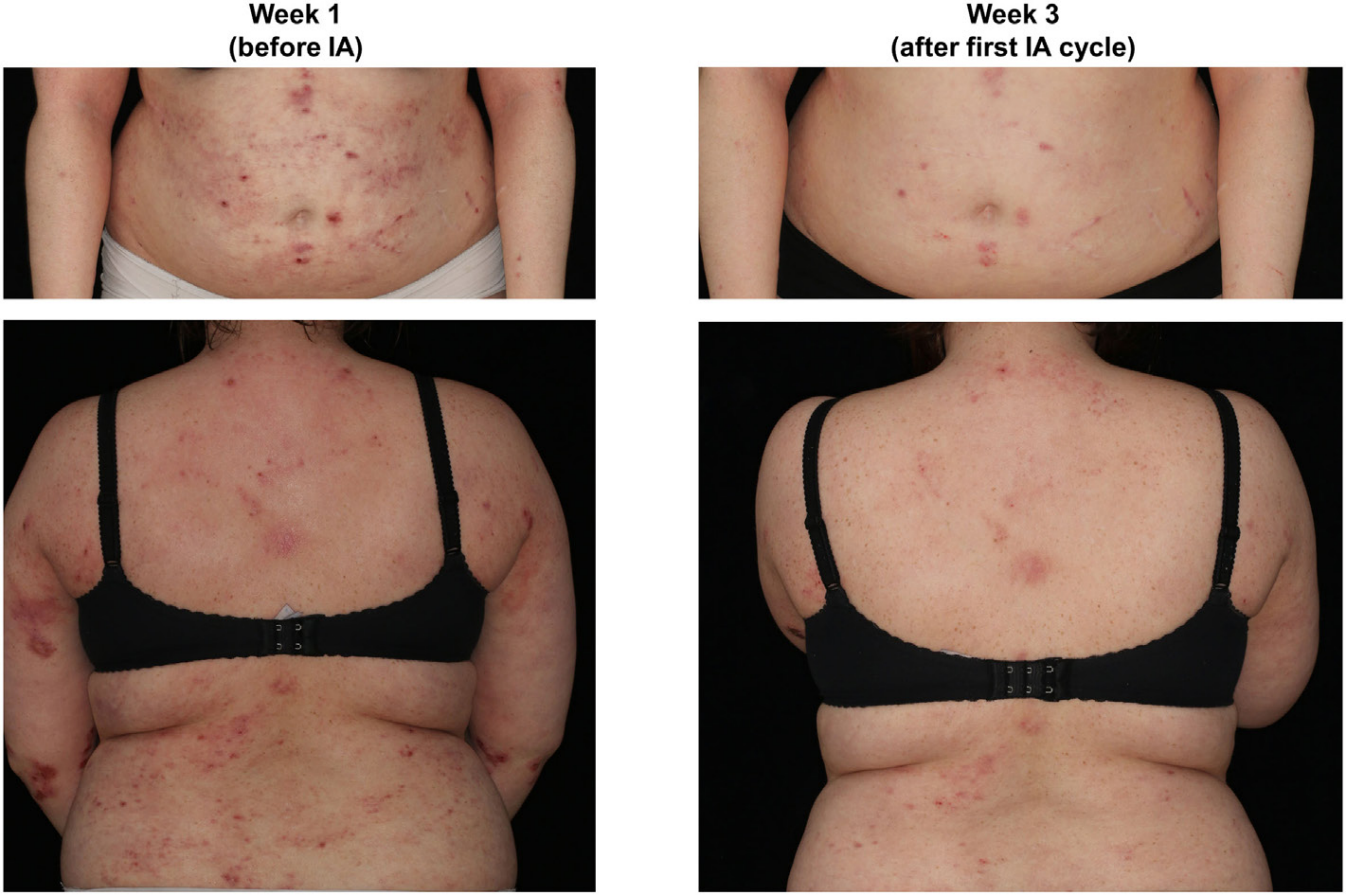
Representative presentation of a study patient before (week 1) and after immunoadsorption (IA) (week 3).

**Figure 2:**
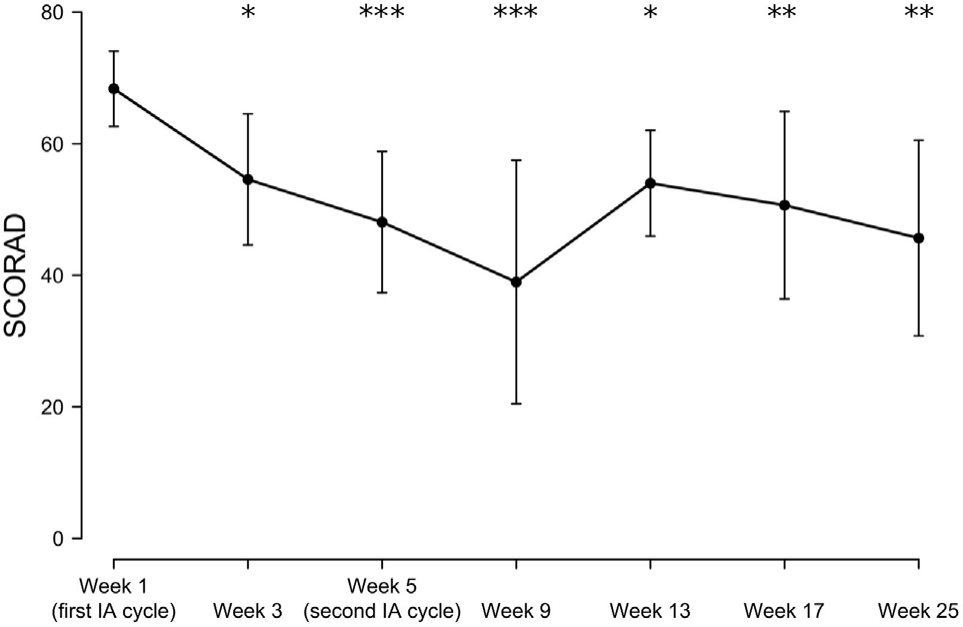
Time-dependent mean SCORAD changes following immunoadsorption (IA). *n* = 3–8 patients (8, 5, 8, 5, 3, 3, and 4 patients at week 1, 3, 5, 9, 13, 17, and 25, respectively). *P < 0.05, **P < 0.01, and ***P < 0.001.

The mean initial SCORAD (68.3 ± 5.7) improved significantly by 19% at week 3 (to a mean score of 54.5 ± 9.9; *p* < 0.05), by 29% at week 5 (to a mean score of 48.0 ± 10.7; *p* < 0.001), by 43% at week 9 (to a mean score of 38.9 ± 18.5; *p* < 0.001), by 21% at week 13 (to a mean score of 54.0 ± 8.0 *p* < 0.05), by 25% at week 17 (to a mean score of 50.6 ± 14.2; *p* < 0.01), and by 29% at week 25 (to a mean score of 48.2 ± 13.1; *p* < 0.01), with individual maximal SCORAD reductions ranging from 15 to 80% and a SCORAD50 response (i.e., an at least 50% reduction of the baseline SCORAD) in 50% of patients. A slight increase in the mean SCORAD was observed in week 13, although by this time and later, only 3–4 patients could be analyzed as the remainder had dropped out or was additionally lost to follow-up (Table 1; Figures 1 and 2).

**Table 1:** Individual SCORAD values of the analyzed study patients.

Pat. no.	Week 1 (1. IA cycle)	Week 3	Week 5 (2. IA cycle)	Week 9	Week 13	Week 17	Week 25
1	69.5	61.0	65.5	58.5	61.5	61.5	61.5
2	66.5	40.2	35.2	26.0	–	–	43.5
3	68.0	62.5	53.0	44.4	45.5	34.5	32.0
4	64.5	61.2	56.1	–	55.0	56.0	56.0
5	69.0	–	39.0	14.0	–	–	–
6	67.0	–	54.6	–	–	–	–
7	61.5	–	43.3	–	–	–	–
8	81.0	48.0	38.0	52.0	–	–	–

*IA, immunoadsorption*.

There were generally no major changes in the use of the previously prescribed topical and systemic medications during the study, except for one patient in whom cyclosporine A (250 mg/day) was reinitiated because of worsening of the disease in week 17.

### Serum Immunoglobulin Levels

**Figure 3:**
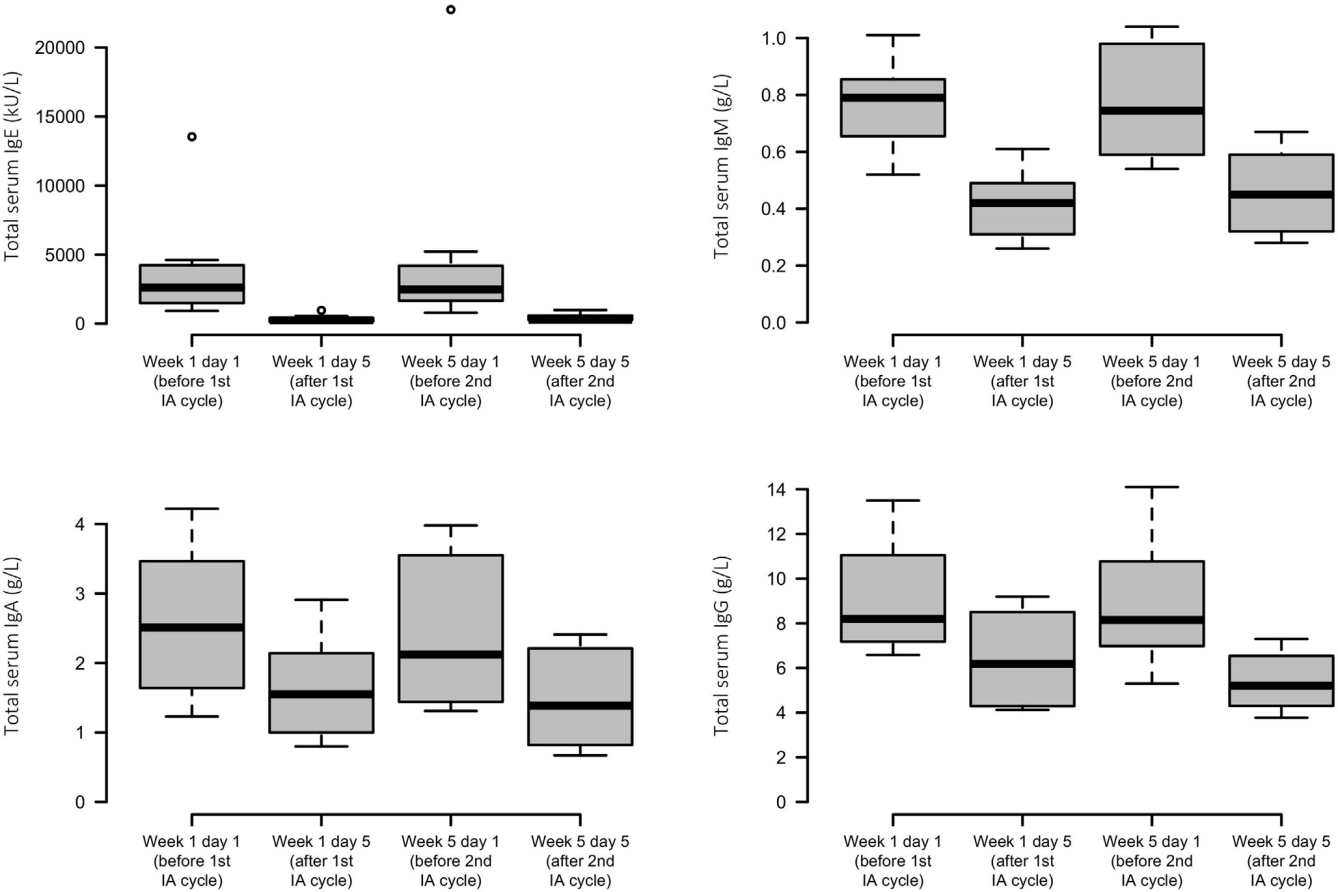
Effects of immunoadsorption (IA) on serum immunoglobulin levels. Box plots of peripheral IgE, IgM, IgA, and IgG levels of the study patients before and after each IA cycle; *n* = 6–8 patients (varying by time point and analyte).

Each IA cycle induced an average decrement of total IgE levels by 92%. Less substantial reductions in IgM, IgA, and IgG were found, with means of 43, 38, and 35%, respectively. Levels of all immunoglobulins were lowered only transiently and reached similar precycle values again (Figure 3).

## Discussion

We recently reported a successful response of a series of treatment-refractory AD patients with excessively high-serum IgE levels to panimmunoglobulin IA and two such patients to IgE-selective IA (5, 6). Using the same treatment protocol of two cycles of five apheresis sessions as in these previous case series, this new study with a further cohort of patients with severe AD and greatly elevated IgE levels extends our knowledge regarding the efficacy of IgE-specific IA. Although somewhat lower mean SCORAD reductions were observed compared with our two preceding reports (e.g., by 19% and 33–38% at week 3 and by 21% and 53–59% at week 13, respectively) (5, 6), the observed clinical improvements were still constantly significant at all examination time points including the last follow-up week 25 compared with baseline.

Recently, an independent head-to-head comparison trial of three cycles of a total of 10 IA sessions of either panimmunoglobulin IA or IgE-selective IA applied over a 2-month period in patients with severe AD with excessive IgE levels revealed that there are no major differences in the positive clinical response between the two different IA treatment groups (9).

Like in our two previous studies (5, 6), serum IgE levels were effectively but transiently reduced by a mean of more than 90% with each IA cycle. In contrast to panimmunoglobulin IA which decreased all other immunoglobulin isotypes to a similar degree (5), serum concentrations of IgM, IgA, and IgG were found to be reduced by only less than half with the IgE-selective adsorber. This latter finding is considered to be nonspecific and has been attributed to IA-related elution and dilution procedures as reported before (6, 9).

Correlation between serum IgE levels and AD severity has been suggested, but study results are partly conflicting (10, 11). A recent study combining panimmunoglobulin IA for 2–4 days with subsequent biweekly omalizumab treatment for 6 months showed that after initial reduction in total serum IgE levels by IA, free IgE levels continued to fall during omalizumab administration and began to increase again during treatment-free follow-up. In addition, in parallel with free IgE levels, an improvement in AD was found during the treatment period, with aggravation during follow-up (8). On the other hand, we and others showed that a prolonged decrease of the SCORAD is not hampered by the commonly seen reincrease in serum IgE that occurs within a relatively short time after IA (5–7, 9). Since in our initial study, an IA-induced continuous reduction of the amount of skin-bound IgE was observed, which correlated with histoimmunological and clinical improvements (5), we speculate that this indirect effect rather than a sole impact of IA on circulating IgE contributes to amelioration of AD. In fact, omalizumab has been previously shown to interfere with antigen processing and presentation to T cells by downregulating IgE-Fcε receptor I expression on dendritic cells (12). Nevertheless, the role of circulating and tissue-bound IgE in the pathogenesis and disease activity of AD as well as the detailed mechanistic interaction of IA with IgE-associated processes in this disease remain in need of further elucidation.

It also remains to be clarified which kind of IA protocol is best suited for AD patients and how long they can ultimately benefit from this treatment. The published trials so far using different IA protocols with follow-up times ranging from 3 to 18 months uniformly revealed a satisfactory initial linear response after treatment (5–9). Results from this current study and our former two reports indicate that IA treatment results in continuous and stable clinical improvements of at least 3–6 months (5, 6), although some minor reincreases in the SCORAD were observed during the second half of the extended observation timeframe (from 3 months in the panimmunglobulin IA trial up to 6 months) in this and our previous IgE-selective IA case series (6). However, this slight worsening in the mean disease severity observed in the current investigation, which was still significantly lower than before initiation of IA, could be at least partly explained by a potential bias due to the relatively high proportion of patients who dropped out or were additionally lost to follow-up during this time period. In fact, the considerable withdrawal rate, the observation that concomitant AD treatment was basically not reduced or discontinued during the study, and the lack of an appropriate control group represent major limitations in judging the effects of adjuvant IA in this patient cohort.

In the study by Reich et al. (9), clinical effects remained stable until 6 months of follow-up in the IgE-selective IA group and slightly reincreased toward the last visit in the panimmunoglobulin arm. As mentioned before, the IA-omalizumab combinatory study revealed a steady decrease in the SCORAD throughout the 6-month treatment period, whereas a reverse trend was observed during follow-up of another 6 months (8). Finally, a study that investigated the efficacy of 1–5 series of panimmunoglobulin IA, each consisting of five consecutive treatments performed on a monthly regimen, revealed no additional benefit with regard to further SCORAD improvement when more than three series were applied. However, one of the patients of this study who completed five IA series exhibited a long lasting clinical benefit over 12 months (7).

A shortcoming of panimmunoglobulin IA is the patient’s potential risk of infections because of the parallel reduction of protective immunoglobulins. In fact, in the study directly comparing unspecific with specific IA in AD, infectious adverse events were limited to the panimmunoglobulin group and comprised herpes labialis/keratitis and bacterial conjunctivitis/sinusitis (9). In contrast, however, a central venous catheter-related *S. aureus* septicemia was observed in both our previous and current study using the nonselective and selective adsorber, respectively (5). Thus, even with IgE-specific IA, a thorough risk-benefit assessment is advised especially for patients in whom a peripheral venous access is not possible, considering that AD is strongly associated with increased susceptibility to skin infections by this bacterial pathogen.

In conclusion, our observations strengthen our and other recent results further suggesting that IgE-selective IA is an effective treatment option for patients severely affected by AD with highly elevated IgE levels. However, given the limitations of this study, including the relatively small number of patients, these results should certainly be considered suggestive rather than definite. In fact, considering that IA has only recently been introduced and that it represents a so far restrictedly used new treatment method for AD, information on efficacy and safety currently still relies on low-level evidence case series. Nevertheless, collection of available data even from these small studies, including the current one, may be important for future meta-analyses or systematic reviews to generate better estimates of the treatment outcomes and may also serve as basis for a potential randomized controlled trial. Thus, future investigations are required to better characterize, among others, the long-term effects and cost–benefit (approximately 6,000 euro for a reusable TheraSorb^®^-IgE adsorber column pair allowing for up to 10 treatment sessions) of IA in AD, for which more optimized treatment protocols still need to be defined.

## Ethics Statement

This study was carried out in accordance with the recommendations of the ethics committee of the University of Lübeck with written informed consent from all subjects. All subjects gave written informed consent in accordance with the Declaration of Helsinki. The protocol was approved by the ethics committee of the University of Lübeck.

## Author Contributions

MK, RL, DZ, and ES contributed to the conception and design of study and analyzed the data. MK, S-CM, DK-R, and PM contributed to the data acquisition. MK, S-CM, DK-R, AV, RL, DZ, PM, and ES interpreted the data, drafted the manuscript, approved the final version of the manuscript, and agreed to be accountable to all aspects of this work. MK, S-CM, DK-R, AV, RL, DZ, PM, and ES approved the final version of the manuscript.

## Conflict of Interest Statement

The authors declare that the research was conducted in the absence of any commercial or financial relationships that could be construed as a potential conflict of interest.
